# Supplementary material for: Influence of acute pain on valence rating of words
Source: PLoS One. 2021 Mar 18;16(3):e0248744. doi: 10.1371/journal.pone.0248744 (PMC7971552; doi:10.1371/journal.pone.0248744)
Supplement: S1 Table — (PDF) [file pone.0248744.s001.pdf]

S1 Table. Valence ratings (mean ± SD) of each word in the main experiment.

Experiment 1 with painful primes

**word**	**mean**	**SD**
**pain-related**		
quälend	7.02	0.35
lähmend	6.89	0.56
zermürbend	6.63	0.65
peinigend	7.47	0.43
plagend	7.19	0.49
kneifend	6.30	0.61
quetschend	6.72	0.56
bohrend	6.32	0.54
kolikartig	6.22	0.67
krampfartig	6.92	0.52
**negative**		
eklig	6.58	0.52
feindlich	7.02	0.78
intrigant	6.92	0.48
widerlich	6.55	0.83
warzig	6.85	0.57
schimmlig	6.73	0.70
stinkend	6.76	0.49
verdreckt	6.57	0.55
angsteinflößend	6.53	0.60
hasserfüllt	7.44	0.40

Experiment 1 without painful primes

**word**	**mean**	**SD**
**pain-related**		
quälend	6.98	0.57
lähmend	6.48	0.82
zermürbend	6.72	0.48
peinigend	6.74	0.75
plagend	6.50	0.64
kneifend	5.79	0.75
quetschend	6.53	0.65
bohrend	6.28	0.73
kolikartig	5.65	0.59
krampfartig	6.41	0.74
**negative**		
eklig	6.81	0.39
feindlich	7.24	0.48
intrigant	6.74	0.50
widerlich	6.41	0.64
warzig	6.54	0.64
schimmlig	6.42	0.65
stinkend	6.61	0.65
verdreckt	5.85	0.56
angsteinflößend	6.36	0.50
hasserfüllt	7.18	0.55

Experiment 2 with painful primes

**word**	**mean**	**SD**
**pain-related**		
quälend	7.65	0.11
lähmend	6.99	0.10
zermürbend	7.15	0.11
peinigend	7.54	0.16
plagend	7.29	0.15
kneifend	6.71	0.20
quetschend	6.93	0.18
bohrend	6.54	0.25
kolikartig	6.81	0.28
krampfartig	7.29	0.20
**negative**		
eklig	6.80	0.25
feindlich	7.42	0.32
intrigant	7.19	0.19
widerlich	7.14	0.21
warzig	7.08	0.21
schimmlig	7.00	0.11
stinkend	6.80	0.12
verdreckt	6.64	0.15
angsteinflößend	7.38	0.36
hasserfüllt	8.04	0.28

Experiment 2 without painful primes

**word**	**mean**	**SD**
**pain-related**		
quälend	7.51	0.15
lähmend	6.73	0.22
zermürbend	6.89	0.10
peinigend	7.39	0.19
plagend	6.97	0.18
kneifend	6.42	0.17
quetschend	6.73	0.22
bohrend	6.27	0.36
kolikartig	6.64	0.26
krampfartig	7.13	0.29
**negative**		
eklig	6.71	0.13
feindlich	7.12	0.31
intrigant	6.93	0.27
widerlich	6.93	0.30
warzig	6.99	0.18
schimmlig	6.82	0.10
stinkend	6.61	0.13
verdreckt	6.49	0.11
angsteinflößend	7.21	0.44
hasserfüllt	7.92	0.31

**neutral**		
gehend	4.68	0.61
eckig	4.98	0.58
kurzhaarig	4.78	0.33
eiförmig	4.65	0.57
gewölbt	4.68	0.58
aschblond	4.86	0.65
klappbar	4.99	0.58
kubisch	4.95	0.48
traubenförmig	4.73	0.59
auditiv	4.76	0.51
**positive**		
streichelnd	3.43	0.51
wärmend	3.03	0.69
erquickend	3.33	0.73
beschwingend	3.78	0.57
himmlich	3.31	0.56
flirtend	3.15	0.64
kuschelnd	3.04	0.64
küssend	2.93	0.66
hocherotisch	3.52	0.63
bezaubernd	3.04	0.61

**neutral**		
gehend	4.61	0.48
eckig	4.65	0.52
kurzhaarig	4.52	0.71
eiförmig	4.61	0.57
gewölbt	4.65	0.64
aschblond	4.77	0.73
klappbar	4.80	0.55
kubisch	4.98	0.68
traubenförmig	4.25	0.56
auditiv	4.18	0.49
**positive**		
streichelnd	2.98	0.66
wärmend	3.11	0.73
erquickend	3.53	0.67
beschwingend	2.97	0.30
himmlich	2.68	0.78
flirtend	3.09	0.60
kuschelnd	2.41	0.54
küssend	2.45	0.48
hocherotisch	3.18	0.58
bezaubernd	2.53	0.78

**neutral**		
gehend	4.56	0.29
eckig	4.99	0.27
kurzhaarig	4.87	0.38
eiförmig	4.88	0.14
gewölbt	4.88	0.32
aschblond	4.50	0.20
klappbar	4.67	0.07
kubisch	4.73	0.23
traubenförmig	4.74	0.31
auditiv	4.33	0.08
**positive**		
streichelnd	2.53	0.24
wärmend	2.53	0.21
erquickend	2.96	0.18
beschwingend	2.95	0.16
himmlich	2.29	0.19
flirtend	2.57	0.25
kuschelnd	2.12	0.19
küssend	2.18	0.25
hocherotisch	3.12	0.17
bezaubernd	2.45	0.20

**neutral**		
gehend	4.42	0.31
eckig	4.91	0.23
kurzhaarig	4.80	0.34
eiförmig	4.83	0.08
gewölbt	4.64	0.10
aschblond	4.41	0.17
klappbar	4.63	0.06
kubisch	4.53	0.13
traubenförmig	4.57	0.11
auditiv	4.20	0.11
**positive**		
streichelnd	2.31	0.26
wärmend	2.36	0.23
erquickend	2.70	0.20
beschwingend	2.83	0.29
himmlich	2.13	0.11
flirtend	2.42	0.18
kuschelnd	1.84	0.16
küssend	1.96	0.21
hocherotisch	2.86	0.20
bezaubernd	2.16	0.14
