# Supplementary material for: Influence of acute pain on valence rating of words
Source: PLoS One. 2021 Mar 18;16(3):e0248744. doi: 10.1371/journal.pone.0248744 (PMC7971552; doi:10.1371/journal.pone.0248744)
Supplement: S2 Table — (PDF) [file pone.0248744.s002.pdf]

S2 Table. Corrected p-values for contrasts regarding interaction effect *Category*Word* in the main experiment.

***: p < 0.001, **: p < 0.01, *: p < 0.05.

pain-related words

	lähmend	zermürbend	peinigend	plagend	kneifend	quetschend	bohrend	kolikartig	krampfartig
quälend	.196	.036*	1.000	.006**	<.001***	.064	<.001***	.006**	.002**
lähmend		1.000	.064	1.000	.029*	1.000	.098	.095	1.000
zermürbend			.004**	1.000	.006**	1.000	.120	.166	1.000
peinigend				<.001***	<.001***	.024*	<.001***	.003**	<.001***
plagend					.001**	1.000	.006**	.058	.132
kneifend						.058	1.000	1.000	.012*
quetschend							.165	.172	1.000
bohrend								1.000	.095
kolikartig									.172

negative words

	feindlich	intrigant	widerlich	warzig	schimmlig	stinkend	verdreckt	angsteinflößend	hasserfüllt
eklig	.007**	1.000	.044*	1.000	1.000	1.000	.026*	1.000	<.001***
feindlich		.416	.250	.430	.430	.016*	<.001***	.017*	1.000
intrigant			1.000	1.000	1.000	1.000	.142	1.000	.002**
widerlich				1.000	1.000	.092	<.001***	1.000	.002**
warzig					1.000	1.000	.024*	1.000	.008**
schimmlig						.142	.003**	1.000	.024*
stinkend							.044*	1.000	<.001***
verdreckt								.016*	<.001***
angsteinflößend									<.001***

neutral words

	eckig	kurzhaarig	eiförmig	gewölbt	aschblond	klappbar	kubisch	traubenförmig	auditiv
gehend	.205	1.000	.477	1.000	1.000	1.000	1.000	1.000	1.000
eckig		1.000	1.000	1.000	1.000	1.000	1.000	.700	.205
kurzhaarig			1.000	1.000	1.000	1.000	1.000	1.000	1.000
eiförmig				1.000	1.000	1.000	1.000	1.000	1.000
gewölbt					1.000	1.000	1.000	1.000	1.000
aschblond						1.000	1.000	1.000	1.000
klappbar							1.000	1.000	1.000
kubisch								.415	.556
traubenförmig									1.000

positive words

	wärmend	erquickend	beschwingend	himmlisch	flirtend	kuschelnd	küssend	hocherotisch	bezaubernd
streichelnd	1.000	1.000	1.000	.395	1.000	.135	.056	.213	1.000
wärmend		1.000	1.000	1.000	.635	1.000	1.000	1.000	.446
erquickend			1.000	.003**	1.000	.408	.131	1.000	.395
beschwingend				.001**	1.000	.395	.147	1.000	.147
himmlisch					.193	1.000	1.000	.006**	1.000
flirtend						.130	.006**	.446	1.000
kuschelnd							1.000	.029*	1.000
küssend								.003**	1.000
hocherotisch									.011*
