# Supplementary material for: Influence of acute pain on valence rating of words
Source: PLoS One. 2021 Mar 18;16(3):e0248744. doi: 10.1371/journal.pone.0248744 (PMC7971552; doi:10.1371/journal.pone.0248744)
Supplement: Attachment — Submitted filename: Response to Reviewers_2021-03-02_Th.docx [file pone.0248744.s004.docx]

**Responses to Journal Requirements and Reviewers**

We thank the reviewers for taking time and effort to provide their constructive comments to improve the manuscript (Ms). We have addressed all the comments in a point-by-point reply. Our answers are indicated in blue font below. We provide a revised manuscript with track changes as well as an unmarked version of the revised manuscript. We report places of changes in this letter with respect to the marked-up copy of Ms.

**Reviewer 1**

The manuscript has been properly improved in many ways: a clearer rationale, the experimental procedure has been better described, a more informative data analysis and results, and the discussion goes more in-depth into the cognitive mechanisms that may be explaining the results.

Thank you.

However, I am afraid that I still cannot recommend the current version for publication because the two main concerns I raised in the first review have not been addressed. First, the AoA of words is still unknown. If there were no AoA scores from previous studies for the words used in the experiments, the authors could have collected them through a rating study, which should not have been complicated given the low number of items to rate. In that case, if the mean AoA scores were similar by categories a possible confounding effect due to AoA of words would have been discarded; if the AoA scores were not similar by categories the speculation about a possible effect of AoA could have been state more exactly and based on data. In any case, I consider that including the omission of AoA scores as a limitation is an easy and not a sufficient solution.

***Answer:***

We followed your advice and conducted a small rating study for the words used in our experiments to explore the AoA. Unfortunately, there is no German database containing the AoA of our adjectives. Birchenough, Davies, & Connelly (2017) delivered norms for over 3,200 German words, but only investigated the AoA of nouns and verbs. We used the same instructions and questionnaire as Birchenough, Davies, & Connelly (2017). We found similar mean AoA scores by categories. Details to this rating study are given in the Overall Discussion (lines 515-525). Furthermore, we adjusted the parts concerning AoA in Limitation and future directions (lines 546-558).

In addition, we searched the AoA norm by Birchenough, Davies, & Connelly (2017) for words similar to the ones we used in our study (with similar word stem) and found similar means to our rating study:

| **Birchenough, Davies, & Connelly (2017)** | |  | **our rating study** | |
| --- | --- | --- | --- | --- |
| **word** | **Mean AoA** |  | **word** | **Mean AoA** |
| Schimmel | 5.9 |  | schimmlig | 6.0 |
| stinken | 3.6 |  | stinkend | 3.7 |
| Dreck | 4.0 |  | verdreckt | 3.3 |
| gehen | 2.9 |  | gehend | 2.8 |
| blond | 4.6 |  | aschblond | 4.4 |
| Himmel | 3.2 |  | himmlisch | 3.4 |
| Zauber | 5.5 |  | bezaubernd | 4.5 |

On the other hand, a by-item analysis has not been carried out as I encouraged to perform. Without it we cannot rule out that the results are due to a few items that generate a strong effect. In other words, the effects are not generalizable to other (similar) words.

***Answer:***

We followed the advice and ran the analyses by item. We found a main effect for the factor *Word* and an interaction effect *Category*Word*. Post-hoc tests are presented in Table S2 in the supporting information. It seems that the words in the positive and neutral categories are rated more homogeneously concerning their overall valence in contrast to the pain-related and negative categories. However, we found no significant interaction between the factors *Word* and *Prime*. This indicates that the effect of the factors *Prime* and *Category* on valence ratings are not just due to a few items generating a strong effect. It rather shows a more general effect not just of individual words, but of word categories. We adjusted the affected parts in the Results and Discussion sections (lines 201/202, 212-217, 234-237, 317-320, 376, 391-398).

**Reviewer 2**

The manuscript has improved a lot by the changes made since the first submission. The authors have taken into account all comments and have responded appropriately to them. I would only like to suggest a few things: In results section: when talking about significant differences found, it is convenient to always indicate in which direction these differences go in the text. Even though there is a figure or the means, it is useful that also indicated in the text. Like in lines 223, 264.

***Answer:***

We thank you for this advice. In accordance with your suggestion, we adjusted the results sections (lines 223/224, 228/229, 252-257, 272/273, 286, 406, 408, 415).
